# Supplementary material for: Temporal trends in African healthcare system capacities in prevention, detection, response, and sustainability between 2010 and 2023
Source: PLOS Glob Public Health. 2026 May 12;6(5):e0005285. doi: 10.1371/journal.pgph.0005285 (PMC13166948; doi:10.1371/journal.pgph.0005285)
Supplement: Attachment — Submitted filename: Response to Reviewers.docx [file pgph.0005285.s023.docx]

**Response to Reviewer 1**

We thank the reviewer for the careful and constructive evaluation of our manuscript. We appreciate the opportunity to clarify methodological decisions, improve transparency in how SPAR indicator evolution was handled, and tighten the interpretation and presentation of results. Below we address each comment point-by-point and specify the exact changes made in the revised manuscript.

**Comment 1:**

Clarify how SPAR data from 2010–2023 were harmonized, given indicator evolution. Please explicitly state how differences in indicator definitions were adjusted or normalized before aggregating domain scores.

**Response:**
We agree that data harmonization detail is essential. We revised the Methods section under *Indicator Evolution and Comparability Over Time* to state that harmonization was conducted at the functional domain level rather than attempting to normalize individual indicators across reporting periods. Specifically, we describe that indicators were mapped to the four domains based on their conceptual role within the IHR framework at the time of reporting, and included only in the years they were defined and available. We also clarify that for each country-year, domain scores were calculated as the mean of all indicators contributing to that domain in that reporting year and then aggregated to regional means. This text addresses how indicator evolution was accommodated prior to aggregation.

**Comment 2:**

With indicator evolution, how did that impact the four functional domains? Consider a table showing when each indicator was added to reflect evolution.

**Response:**
To address this, we added **Table 4**: *“SPAR indicator composition by functional domain and reporting period (2010–2023)”*. Table 4 shows the indicator sets used in each reporting period (2010–2017, 2018–2020, 2021–2023) and their assignment to Prevention, Detection, Response, and Sustainability. I believe this this provides a structured depiction of how domain composition evolved over time.

**Comment 3:**

Averaging domain indicators (rather than weighting by importance or completeness) may bias trends. Was any sensitivity analysis conducted?

**Response:**
We acknowledge this concern and revised the Methods section under *Domain score construction and weighting considerations* to justify unweighted averaging. We state that equal weighting was selected to avoid subjective assumptions about relative indicator importance given periodic revisions in SPAR definitions and scope, and because SPAR indicators are ordinal and designed to reflect complementary aspects of preparedness. We also explain that formal sensitivity analyses using alternative weighting schemes were not performed, because reliable external criteria to justify differential weighting across indicators and reporting periods were not available. We clarify that trends are interpreted at the domain level rather than individual indicators.

**Comment 4:**

ANOVA and Tukey HSD are appropriate, but confirm assumptions (normality, homogeneity of variance) were met.

**Response:**
We added a statement in the Methods section under *Assessment of regional differences* confirming that data distributions were assessed for approximate normality and homogeneity of variance using standard diagnostic checks, and that no substantial violations were identified that would preclude parametric testing, supporting the use of ANOVA and Tukey HSD.

**Comment 5:**

Linear regression is reported, but R² indicates only moderate explanatory power. Add discussion on potential non-linearity or whether other time-series models were tested.

**Response:**
We revised the Results and Discussion to interpret linear regression appropriately. In the Results (*Trend analysis from 2010 to 2023*), we now clearly state that moderate R² values indicate linear trends explain only a portion of observed variance, reflecting temporal changes and potential non-linear shifts. In the Discussion, we added a paragraph stating that trajectories were not strictly linear, that several domains show inflection points around COVID-19, and that non-linear or segmented time-series approaches were not applied because the objective was to note overall directionality and relative differences rather than model short-term changes. This directly addresses the reviewer’s concern about explanatory power and non-linearity.

**Comment 6:**

Include confidence intervals for regression coefficients to align with BMC reporting standards.

**Response:**
We added 95% confidence intervals for regression coefficients directly into **Table 2**: *Linear regression estimates of temporal trends in SPAR functional domain scores by region, 2013–2023,* which reports β (slope), 95% CI, R², and p-values for each domain and region. We also reference **Table 2** in the Results regression section.

**Comment 7:**

Comment on regional differences; add more description (e.g., income groups).

**Response:**
We added regional context in two places. First, we inserted an explicit contextual statement at the start of the Results noting that African regions differ in baseline health system structure and economic capacity, with Northern Africa comprising predominantly upper-middle-income countries and other regions including higher proportions of low- and lower-middle-income countries. Second, we expanded the Discussion to interpret regional differences in relation to structural and economic differences (per-capita income, infrastructure, workforce shortages, dependence on external financing), with supporting citations.

**Comment 8:**

Several Results statements would benefit from quantitative qualifiers (absolute/relative change).

**Response:**
We revised the Results to include explicit quantitative qualifiers, including absolute scores and relative percent changes, across domains and time points (2013, 2018, 2023), and particularly for pre/post COVID comparisons. Examples now include statements such as Africa’s response capacity increasing from 38.48 (2018) to 59.86 (2023) with ~55% improvement, and similar quantified changes for prevention, detection, and sustainability across global and regional comparisons.

**Comment 9:**

Clarify what “sustainability” captures conceptually.

**Response:**
We added a conceptual definition directly in the Methods under *Data source and capacity domains*, stating that sustainability reflects the capacity to maintain essential public health functions over time independent of acute response, including financing stability, workforce retention, service continuity, and supply chain resilience, emphasizing durability beyond episodic shocks. This clarifies the construct for readers unfamiliar with SPAR.

**Comment 10:**

The Discussion could be tightened to focus on empirical implications.

**Response:**
We revised the Discussion to reduce redundancy and maintain emphasis on interpretation of the empirical results, including consolidating overlapping statements and focusing domain-specific interpretation (prevention, detection, response, sustainability) and the quantified pre/post COVID comparisons.

**Comment 11:**

Add a short policy paragraph summarizing actionable regional priorities.

**Response:**
We added a dedicated policy-oriented paragraph in the Discussion beginning “These findings suggest region-specific priorities…”, specifying actionable priorities by region (foundational surveillance/lab capacity in Middle Sub-Saharan Africa; workforce retention/financing stability and integration of emergency gains in Western/Eastern Sub-Saharan Africa; sustaining investments and cross-border coordination in Northern Africa).

**Comment 12:**

Tables 1 and 2 should include sample size (n countries) per region and year.

**Response:**
We revised **Table 1** to clearly indicate that n represents sample size per region and year, and revised Table 2, now **Table 3**, to state that the total number of reporting countries is listed in the rightmost column for each region.

**Comment 13:**

Figures 2–3: Add error bars or 95% CI bands.

Response:
We updated the figure legends to reflect uncertainty visualization: **Figure 2** now specifies that error bars represent standard deviation, and **Figure 3** specifies that 95% CI bands are depicted with dotted lines, alongside p-values for each regional trend.

**Comment 14:**

Shorten the Discussion and tone down the conclusion.

**Response:**
We tightened the Discussion to reduce overlap and refined the Conclusion to be non-prescriptive. The revised Conclusion is limited to two sentences and frames implications cautiously (“may be warranted”) rather than making strong prescriptive claims.
