# Supplementary material for: Temporal trends in African healthcare system capacities in prevention, detection, response, and sustainability between 2010 and 2023
Source: PLOS Glob Public Health. 2026 May 12;6(5):e0005285. doi: 10.1371/journal.pgph.0005285 (PMC13166948; doi:10.1371/journal.pgph.0005285)
Supplement: Attachment — Submitted filename: Response_to_Reviewers_auresp_2.docx [file pgph.0005285.s025.docx]

**Response to Reviewers**

*Manuscript: Temporal trends in African healthcare system capacities in prevention, detection, response, and sustainability between 2010 and 2023*

We thank both reviewers for their thorough and constructive evaluation of our revised manuscript. We have carefully addressed each comment and describe the specific changes made below. All revisions are indicated with track changes in the revised manuscript. Page and line numbers refer to the revised manuscript.

**Response to Reviewer #1:**

We thank Reviewer 1 for the positive assessment of the revised manuscript.

**Comment 1:**

*The manuscript has improved a lot. I still recommend making the discussion more concise and improving the flow. i.e. move the limitations towards the end of the discussion rather than early on.*

**Response:**

We agree with this structural recommendation. The **Limitations and Data Considerations** section has been moved from its previous position early in the Discussion to near the end, now appearing at lines 651–700 of the revised manuscript, after all domain-specific interpretation paragraphs and the regional priorities paragraph, and immediately before the Conclusion. We also reviewed the Discussion for redundant content and tightened language throughout to improve flow.

**Response to Reviewer #2:**

We thank Reviewer 2 for the detailed and constructive critique. We address each major and minor comment in turn below.

**Major Comment 1: Comparability Across SPAR Reporting Frameworks**

*The manuscript appropriately notes that SPAR indicators evolved across reporting periods (2010–2017, 2018–2020, and 2021–2023). However, the implications of these structural changes for longitudinal analysis are not fully explored. The observed decline around 2018 may partly reflect revisions in the reporting framework rather than a true decrease in preparedness capacity. Without sensitivity analyses or formal testing for structural breaks, it is difficult to disentangle measurement effects from real trends.*

**Response:**

We agree this is an important concern and have addressed it with a formal sensitivity analysis. We performed linear regression restricted to the 2018–2023 period for all four domains and seven regional groups, encompassing a single continuous SPAR reporting framework. Results are presented in the newly added **Supplementary Table S1**.

Specific changes in the revised manuscript are as follows:

**Methods, Indicator Evolution and Comparability Over Time (lines 218–228):** Added to the final paragraph of this section: “Of the three reporting transitions, the shift from the 2017 to 2018 framework involved the most substantial restructuring in indicator composition, including the addition, removal, and redefinition of multiple indicators across all four domains (Table 4). Observed score changes around this transition may therefore partly reflect measurement revision rather than true changes in underlying preparedness capacity. To address this, a sensitivity analysis was performed restricting regression analyses to the 2018–2023 period; results are described in the Results and S1 Table.”

**Methods, Trend analysis using regression modeling (lines 304–311):** Added a new dedicated paragraph: “As a sensitivity analysis to assess the potential impact of the 2018 SPAR framework transition on observed trends, linear regression was additionally performed restricting the analysis to the 2018–2023 period. This restricted analysis encompasses a single continuous reporting framework and avoids the structural discontinuity introduced at the 2017–2018 transition. Results from both the full-period and restricted analyses are presented in S1 Table and compared to assess directional consistency and robustness of the reported trends.”

**Results, Trend analysis from 2010 to 2023 (lines 436–480):** The Results trend analysis section was substantially revised. Full-period regression findings are now reported with explicit slopes and p-values (lines 436–448). Three new paragraphs (lines 450–480) report the sensitivity analysis results in detail, including the directional reversal of prevention trends, the emergence of significant post-2018 response and sustainability trajectories, and the plateauing of detection gains post-2018.

**Discussion, opening paragraph (lines 504–518):** Added a dedicated paragraph reporting how sensitivity analyses clarified all four domain trends, interpreting the prevention reversal as driven by the 2018 framework transition rather than genuine deterioration.

**Limitations and Data Considerations (lines 681–700):** Added a new paragraph explicitly acknowledging that formal structural break testing was not performed and that the sensitivity analyses should be considered partial rather than definitive.

**Major Comment 2: Analytical Strategy (Regression and Aggregation)**

*The study uses OLS regression on annual regional means (approximately 14 time points). While this provides a high-level overview, this approach masks country-level variability, does not explicitly account for the panel nature of the data, and does not address potential serial correlation across time. If re-analysis is not feasible, I recommend clearly justifying the chosen approach and discussing its limitations more explicitly in the Methods and Discussion sections.*

**Response:**

Re-analysis using a panel modeling approach was not feasible within the scope of this revision. We have followed the reviewer’s recommended alternative of clearly justifying the chosen approach and discussing its limitations explicitly.

**Methods, Trend analysis using regression modeling (lines 293–302):** Added the following paragraph immediately after the existing OLS description: “Regional mean-level regression was selected because the primary unit of analysis in this study is the regional aggregate rather than the individual country. This approach provides a transparent and interpretable summary of continent-wide and regional-level preparedness trajectories consistent with the study’s descriptive aims. It does not account for the panel structure of the underlying country-year data, serial correlation across time points, or within-region heterogeneity; a fixed or random effects panel model would be more appropriate for country-level inference, which was not the objective of this analysis.”

**Limitations and Data Considerations (lines 673–679):** Added: “Similarly, the regression analyses were conducted on annual regional means rather than on country-year panel data. This approach was intentional given the study’s regional-level descriptive aims, but it does not account for serial correlation across time points or within-region country-level variability. Findings from the regression analyses should therefore be interpreted as characterizing broad regional trajectories rather than precise estimates of trend magnitude.”

**Major Comment 3: Interpretation of COVID-19 Effects**

*The manuscript suggests that improvements particularly in response capacity after 2018 may reflect the impact of COVID-19. While this interpretation is plausible, it is not formally tested. Without an interrupted time-series or segmented regression design, causal attribution to COVID-19 is not supported by the current analysis. I suggest reframing conclusions to describe temporal associations rather than causal effects.*

**Response:**

We agree and have reframed all causal language throughout the manuscript. Specific changes are as follows:

**Abstract (lines 67–68):** “Response capacity increased sharply in Africa after COVID-19” changed to “Response capacity increased sharply in Africa in the post-pandemic period.”

**Introduction (lines 134–136):** “The COVID-19 pandemic has pressured African countries’ healthcare systems, exposing weaknesses and highlighting areas for potential improvement” replaced with: “The COVID-19 pandemic coincided with heightened scrutiny of African countries’ healthcare systems, revealing existing weaknesses and drawing attention to areas requiring investment and improvement.”

**Results subheading (line 375):** “Response capacity before and after COVID-19” changed to “Response capacity: regional trends and post-pandemic changes.”

**Results subheading (line 426):** “Effect of COVID-19 on capacity levels (2018–2023)” changed to “Capacity level changes in the post-pandemic period (2018–2023).”

**Results, Response capacity paragraph (lines 386–388):** Added the clause “coinciding with the post-COVID-19 pandemic period during which many countries substantially scaled up emergency preparedness investments” to describe the 2018–2023 improvement without implying causation.

**Discussion, opening paragraph (lines 523–530):** Added explicit causal disclaimer: “It should be noted that without a formal interrupted time-series or segmented regression design, causal attribution of these improvements to the COVID-19 pandemic cannot be established from the current analysis. The observed gains in response capacity are temporally associated with the pandemic period and may reflect heightened emergency preparedness investments during this time, but other concurrent factors cannot be excluded.”

**Discussion, COVID comparison paragraph (lines 611–619):** “The pre- and post-COVID-19 comparison” changed to “The pre- and post-pandemic comparison”; “the pandemic catalyzed improvements” replaced with “the post-pandemic period coincided with improvements”; causal language in detection sentence replaced with temporal association framing.

**Major Comment 4: Self-Reported Data and Reporting Bias**

*SPAR data are self-reported, and this introduces the possibility of reporting bias particularly in the post-pandemic period when preparedness metrics have gained political visibility. Although the manuscript acknowledges this limitation, the discussion could be expanded to reflect how such bias may influence upward trends in later years. At minimum, clarifying that the results reflect perceived rather than independently validated preparedness would improve interpretative clarity.*

**Response:**

We have expanded the treatment of this limitation in three places.

**Abstract (line 49):** “this analysis evaluated national capacities” changed to “this analysis evaluated self-reported national capacities.”

**Results, opening paragraph (line 333):** “observed regional differences in preparedness scores” changed to “observed regional differences in self-reported preparedness scores.”

**Limitations and Data Considerations (lines 653–668):** Added “and results should be interpreted as reflecting perceived rather than independently validated preparedness capacity” to the opening sentence (line 655–656). Added the following new passage (lines 661–668): “This concern is particularly relevant for the post-2020 data, during which preparedness metrics gained considerable political visibility in the context of the COVID-19 pandemic. Upward reporting pressure in this period may have contributed to the observed gains in response and sustainability scores between 2018 and 2023, and findings from this period should be interpreted with this in mind. Triangulation with externally validated preparedness indices such as the JEE or Global Health Security Index was not performed and represents an important direction for future work.”

**Major Comment 5: Regional Aggregation and Within-Region Variability**

*Regional averages provide useful summaries but may obscure important within-region heterogeneity. The manuscript does not present measures of dispersion or variability across countries. Including standard deviations, interquartile ranges, or supplementary variability plots would help readers better understand how evenly (or unevenly) preparedness improvements are distributed within regions.*

**Response:**

Standard deviations were already presented visually as error bars in Figure 2. We have now formally integrated this information into the text and added **Supplementary Table S2** presenting regional mean ± SD for all four domains at 2013, 2018, and 2023.

**Methods, Score calculation and regional aggregation (lines 247–249):** Added: “Regional standard deviations were calculated alongside means to characterize within-region variability in country-level scores and are presented in Figure 2 and S2 Table.”

**Results, Regional progress and stagnation across the decade (lines 420–424):** Added: “Within-region variability was also considerable, particularly in Eastern and Western Sub-Saharan Africa, where standard deviations around regional means were largest, indicating that aggregate scores mask meaningful heterogeneity in individual country performance within these regions (Figure 2, S2 Table).”

**Discussion, regional differences paragraph (lines 545–549):** Added: “Regional averages for these subregions therefore represent central tendency rather than uniform performance, and individual country trajectories may diverge considerably from the aggregate trend. Country-level means and standard deviations for all regions and time points are provided in S2 Table.”

**Responses to Minor Comments:**

**Minor Comment 1: The methodology and discussion sections need to be improved.**

**Response:**

The Methods and Discussion sections were substantially revised in addressing the major comments above. A summary of new additions by section and line number is provided in the responses to Major Comments 1–5 above. The Discussion was additionally restructured with the Limitations section relocated from its prior position to lines 651–700.

**Minor Comment 2: The five-point preparedness classification scale appears somewhat arbitrary; please provide justification or consider removing it.**

**Response:**

**Methods, Pre-pandemic and post-pandemic comparison (lines 271–272):** Added the clause “consistent with ordinal capacity level frameworks used in WHO IHR monitoring” with supporting citation (reference 10: Kandel et al., 2019) to the sentence describing the five-point scale. This citation supports the use of an ordinal classification approach for categorical interpretation of SPAR-based preparedness data.

**Minor Comment 3: Clarify the consistency between the stated study period (2010–2023) and analyses emphasizing 2013 onward.**

**Response:**

**Methods, Data source and capacity domains (lines 176–181):** Added the following clarifying sentence: “Although SPAR data were available from 2010 onward and are included in the regression trend analyses, cross-sectional comparative analyses and ANOVA-based regional comparisons use 2013 as the earliest time point, reflecting the first year with complete domain-level data across all five regions.”

**Abstract (line 51):** “from 2010 to 2023” changed to “from 2013 to 2023” to align with comparative analysis coverage.

**Table 2 header:** Corrected from “2013–2023” to “2010–2023” to accurately reflect the regression period.

**Minor Comment 4: Consider adjusting for multiple comparisons in the ANOVA analyses.**

**Response:**

**Methods, Assessment of regional differences (lines 260–263):** Added the following sentence: “No additional correction for multiple comparisons beyond Tukey’s HSD was applied, as this procedure inherently controls the family-wise error rate across all pairwise regional comparisons.” Tukey’s HSD was selected specifically for this purpose.

**Minor Comment 5: Report confidence intervals alongside percent changes where possible.**

**Response:**

We added three new supplementary tables: **S3, S4, and S5 Tables**. These present pairwise ANOVA comparisons for 2013, 2018, and 2023, respectively, each including mean differences, 95% confidence intervals, and Tukey-adjusted p-values for all key regional comparisons. Regression slope confidence intervals were already reported in **Table 2**.

**Methods, Software and data processing (lines 320–324):** Added: “Percent changes are reported descriptively alongside absolute score differences; 95% confidence intervals for pairwise mean differences from ANOVA are reported in S3–S5 Table, and regression slope confidence intervals are reported in Table 2 and S1 Table.”

**Minor Comment 6: To improve reproducibility, consider providing analytical code as supplementary material.**

**Response:**

All analyses were performed using GraphPad Prism version 11, a GUI-based platform that does not generate exportable code. To maximize reproducibility, we have provided the complete GraphPad Prism project file as **S15 Dataset**, containing all primary analyses with all regional domain score datasets embedded. This allows any reader with GraphPad Prism to verify and reproduce all results directly.

**Methods, Software and data processing (lines 318–320):** Added: “The complete GraphPad Prism project file containing all analyses is provided as S15 Dataset. Original national datasets are additionally available as S1–S14 Datasets.”

We believe these revisions have substantially strengthened the manuscript’s methodological transparency, interpretive accuracy, and reproducibility. We are grateful to both reviewers for their constructive engagement and are happy to address any remaining questions.
