# Supplementary material for: Temporal trends in African healthcare system capacities in prevention, detection, response, and sustainability between 2010 and 2023
Source: PLOS Glob Public Health. 2026 May 12;6(5):e0005285. doi: 10.1371/journal.pgph.0005285 (PMC13166948; doi:10.1371/journal.pgph.0005285)
Supplement: Attachment — Submitted filename: Recommendation- Accept with Minor Revisions.docx [file pgph.0005285.s024.docx]

**Recommendation: Accept with Minor Revisions**

The revised manuscript demonstrates substantial improvement and adequately addresses the major methodological concerns raised in the initial review. In particular, the authors have:

- Clarified the implications of SPAR framework changes and incorporated sensitivity analyses (2018–2023) to improve temporal comparability
- Appropriately reframed interpretations of post-pandemic trends to avoid unsupported causal inference
- Justified the use of regional aggregation and clearly delineated the limitations of the analytical approach
- Expanded the discussion of self-reported data bias, including its potential impact on post-pandemic estimates
- Incorporated measures of within-region variability, improving interpretability of regional summaries

While additional analyses (e.g., structural break testing, panel models, or triangulation with external indices) would further strengthen the work, their absence does not undermine the validity of the current findings. These limitations are now transparently acknowledged and appropriately contextualized.

The manuscript now meets the standards of methodological transparency, analytical coherence, and policy relevance expected for publication. The conclusions are appropriately cautious and aligned with the presented evidence.

**I recommend acceptance pending minor editorial refinements.**
