# Supplementary material for: Temporal trends in African healthcare system capacities in prevention, detection, response, and sustainability between 2010 and 2023
Source: PLOS Glob Public Health. 2026 May 12;6(5):e0005285. doi: 10.1371/journal.pgph.0005285 (PMC13166948; doi:10.1371/journal.pgph.0005285)
Supplement: Attachment — Submitted filename: Reviewer Comments to the Authors.docx [file pgph.0005285.s022.docx]

**Reviewer Comments to the Authors**

**Manuscript Title:**
Temporal trends in African healthcare system capacities in prevention, detection, response, and sustainability between 2010 and 2023

**Summary**

This manuscript presents an important and timely analysis of longitudinal trends in WHO SPAR preparedness capacities across 54 African countries from 2010 to 2023. By grouping countries into five UN-defined regions and examining prevention, detection, response, and sustainability domains, the study offers valuable continent-wide insights into preparedness patterns and regional differences.

The topic is highly relevant, particularly in the post-COVID-19 context. The use of a comprehensive, multi-country dataset over more than a decade is a notable strength. However, there are several methodological and analytical issues that should be addressed to strengthen the validity and interpretability of the findings.

Overall, I believe the manuscript has strong potential, but it would benefit from substantial revision.

**Major Comments**

**1. Comparability Across SPAR Reporting Frameworks**

The manuscript appropriately notes that SPAR indicators evolved across reporting periods (2010–2017, 2018–2020, and 2021–2023). However, the implications of these structural changes for longitudinal analysis are not fully explored. The observed decline around 2018 may partly reflect revisions in the reporting framework rather than a true decrease in preparedness capacity. Without sensitivity analyses or formal testing for structural breaks, it is difficult to disentangle measurement effects from real trends.

I encourage the authors to consider:

- Conducting sensitivity analyses (e.g., restricting to indicators that remained consistent across periods or focusing on the post-2018 timeframe), and/or
- Performing a structural break analysis to assess the impact of framework revisions.

Clarifying this issue would significantly improve confidence in the reported temporal trends.

**2. Analytical Strategy (Regression and Aggregation)**

The study uses OLS regression on annual regional means (approximately 14 time points). While this provides a high-level overview, this approach:

- Masks country-level variability,
- Does not explicitly account for the panel nature of the data, and
- Does not address potential serial correlation across time.

Given that country-year data are available, a panel modeling approach (e.g., fixed or random effects) would provide a more robust analytical framework.

If re-analysis is not feasible, I recommend clearly justifying the chosen approach and discussing its limitations more explicitly in the Methods and Discussion sections.

**3. Interpretation of COVID-19 Effects**

The manuscript suggests that improvements particularly in response capacity after 2018 may reflect the impact of COVID-19. While this interpretation is plausible, it is not formally tested. Without an interrupted time-series or segmented regression design, causal attribution to COVID-19 is not supported by the current analysis.

I suggest either:

- Reframing conclusions to describe temporal associations rather than causal effects, or
- Conducting segmented regression with a clearly defined interruption point (e.g., 2020).

This adjustment would help align the conclusions more closely with the analytical evidence.

**4. Self-Reported Data and Reporting Bias**

SPAR data are self-reported, and this introduces the possibility of reporting bias particularly in the post-pandemic period when preparedness metrics have gained political visibility. Although the manuscript acknowledges this limitation, the discussion could be expanded to reflect how such bias may influence upward trends in later years.

If possible, triangulating findings with external preparedness indices (e.g., Joint External Evaluation or other global metrics) would strengthen credibility. At minimum, clarifying that the results reflect perceived rather than independently validated preparedness would improve interpretative clarity.

**5. Regional Aggregation and Within-Region Variability**

Regional averages provide useful summaries but may obscure important within-region heterogeneity. The manuscript does not present measures of dispersion or variability across countries.

Including standard deviations, interquartile ranges, or supplementary variability plots would help readers better understand how evenly (or unevenly) preparedness improvements are distributed within regions.

**Minor Comments**

- The methology and discussion sections need to be improved
- The five-point preparedness classification scale appears somewhat arbitrary; please provide justification or consider removing it.
- Clarify the consistency between the stated study period (2010–2023) and analyses emphasizing 2013 onward.
- Consider adjusting for multiple comparisons in the ANOVA analyses.
- Report confidence intervals alongside percent changes where possible.
- To improve reproducibility, consider providing analytical code as supplementary material.

**Recommendation**

**Major Revision**

This manuscript addresses an important and policy-relevant topic and has clear potential for publication. However, strengthening the analytical framework and moderating certain interpretations will substantially enhance the robustness and credibility of the findings.
